# Quechua translation and validity of the revised mental health inventory in Peruvian adolescents

**DOI:** 10.3389/fpsyg.2026.1967442

**Published:** 2026-10-12

**Authors:** Jonatan Baños-Chaparro, Miluska Glamis Moreyra-Ruiz, Andrei Franco-Jimenez, Diego Valencia-Pecho, Paul Ynquillay-Lima, Tomás Caycho-Rodríguez

**Affiliations:** 1 Programa académico de Psicología, Facultad de Ciencias de la Salud, Universidad Privada Norbert Wiener, Lima, Peru; 2 Escuela de Posgrado, Universidad Ricardo Palma, Lima, Peru; 3 Facultad de Psicología, Universidad Nacional San Luis Gonzaga, Lima, Peru; 4 Facultad de Psicología, Universidad Autónoma del Perú, Lima, Peru; 5 Instituto de Investigación, Universidad para el Desarrollo Andino, Huancavelica, Peru; 6 Facultad de Psicología, Universidad Científica del Sur, Lima, Peru

**Keywords:** adolescent, indigenous peoples, mental health, Quechua, translation

## Abstract

**Introduction:**

Adolescent mental health is a growing public health concern, particularly among Indigenous populations, where linguistic and cultural barriers may limit access to appropriate psychological assessment. Therefore, having brief and culturally adapted instruments in Indigenous languages is essential to improve mental health assessment and research among Quechua-speaking adolescents.

**Objective:**

To translate and culturally adapt the Revised Mental Health Inventory-5 (R-MHI-5) from Spanish into Southern Quechua and examine its psychometric properties in Peruvian adolescents.

**Methods:**

A total of 2,978 adolescents from Angaraes, Huancavelica, participated in this instrumental, quantitative, cross-sectional study. Participants completed the Southern Quechua version of the R-MHI-5 and a sociodemographic questionnaire. Structural equation modeling and item response theory were applied.

**Results:**

The Southern Quechua R-MHI-5 showed adequate content validity (Aiken’s *V* > 0.70) and a two-dimensional structure comprising psychological well-being and psychological distress, with satisfactory fit indices (CFI = 0.99, RMSEA = 0.03, SRMR = 0.01). Reliability was adequate for both dimensions (*ω* = 0.72; *H* = 0.78), and measurement invariance by sex was supported. Item response theory analyses showed satisfactory item functioning. Item 4 showed the highest discrimination and information for psychological well-being, whereas item 3 showed the highest discrimination and information for psychological distress. The scale provided greater information at intermediate levels of well-being and at intermediate-to-high levels of psychological distress.

**Conclusion:**

The Southern Quechua version of the R-MHI-5 showed adequate psychometric properties supporting its use to assess mental health among Quechua-speaking adolescents. Its brief format, two-dimensional structure, reliability, measurement invariance, and item-level performance support its application in research and mental health assessment in community settings.

## Introduction

Mental health is a growing global challenge with important implications for population wellbeing and overall health (Bajbouj et al., 2022). Accordingly, this construct is defined as a state of mental well-being that enables individuals to develop the capacities needed to cope with the stresses of everyday life, maintain functional and productive behaviors, and contribute to social activities (World Health Organization [WHO], 2022a). Thus, mental health is not merely the absence of mental disorders; rather, the exercise of these capacities constitutes a form of well-being in itself, regardless of the presence of illness or even unfavorable social conditions (WHO, 2022b).

However, this well-being can be disrupted by the presence of mental health conditions, and adolescents represent a particularly vulnerable population group (Moreta-Herrera et al., 2025). Adolescence is a critical developmental period characterized by physical, emotional, social, and cognitive changes that may increase vulnerability to psychological distress (Baños-Chaparro et al., 2024). Likewise, coping with stressors such as developing a sense of identity, becoming less dependent on parental support, pursuing formal education, experiencing academic pressure, and establishing relationships with peers may negatively affect psychological well-being and may even have consequences that persist into adulthood (Jobin et al., 2025).

Globally, common mental health problems among adolescents include anxiety, depressive, and behavioral disorders (Kumbet et al., 2023). In this regard, one in seven adolescents experiences a mental disorder, which in turn accounts for 15% of the total burden of disease and mortality (WHO, 2024). In Peru, adolescent mental health has also been affected by increasing levels of depression and anxiety (Ministry of Health [MINSA], 2025). At the national level, 17.2% presented the highest prevalence of depressive episodes during 2024. Furthermore, distribution by natural region showed a higher prevalence in the Highlands (13.9%) compared with the Coast (9.7%) and the Jungle (8.6%), with even higher levels reported in some provinces of the southern highlands, such as Huancavelica (25.4%), Puno (19.2%), and Cusco (16%) (National Institute of Statistics and Informatics [INEI], 2025).

Given this context, it is important to address the various factors that protect mental health during adolescence (Tinsae et al., 2024). In this regard, the literature identifies variables such as socioemotional skills, self-esteem, autonomy, resilience, and perceived social support which have been associated with higher levels of psychological well-being (Cherewick et al., 2023). Social determinants also play an important role in mental health outcomes because they encompass the circumstances in which individuals are born and develop (WHO, 2024). However, these circumstances vary across different groups of adolescents depending on region, ethnicity, culture, and socioeconomic conditions in which they live (Serrano-Ccopa, 2024). Therefore, sociodemographic differences influence the way adolescents experience mental health (Baños-Chaparro and Ynquillay-Lima, 2023; López and Laviana, 2024).

Quechua is not a single homogeneous linguistic variety but a language family comprising several geographically differentiated varieties (Cjuno et al., 2021). In Peru, Quechua varieties are officially grouped into four major branches: Amazonian Quechua, Northern Quechua, Central Quechua, and Southern Quechua. Southern Quechua comprises two main varieties, Chanka and Collao. Chanka Quechua is spoken mainly in Huancavelica, Ayacucho, and parts of Apurímac, whereas Collao Quechua is spoken mainly in Cusco,

Puno, Arequipa, Moquegua, and other parts of Apurímac (Ministry of Culture of Peru, 2026; Ministry of Education of Peru [MINEDU], 2021). Angaraes, Huancavelica, where the present study was conducted, is located within the geographical area associated with Chanka Quechua. Although Chanka and Collao belong to the Southern Quechua branch and share important linguistic features, differences in phonology and vocabulary exist. Therefore, the psychometric evidence obtained in the present study should not be automatically generalized to speakers of other Quechua varieties.

Regarding mental health assessment, the literature highlights the importance of conceptualizing this construct in terms of two related but distinct dimensions: psychological well-being and psychological distress (Magalhães, 2024). The positive dimension encompasses emotional and psychological well-being and positive affect, whereas the negative dimension includes indicators of psychological distress (Greenspoon and Saklofske, 2001). Within this conceptual framework, the 38-item Mental Health Inventory (MHI) was originally developed in English by Veit and Ware (1983) to assess psychological well-being and psychological distress. Subsequently, Berwick et al. (1991) developed the five-item Mental Health Inventory (MHI-5), an abbreviated version designed to provide a brief assessment of mental health. The original MHI-5 uses six response categories and includes two items assessing psychological well-being and three items assessing psychological distress. Rivera-Riquelme et al. (2019) later translated and adapted the MHI5 into Spanish for children and adolescents and introduced a simplified four-point response format. This revised version, referred to as the R-MHI-5, retained the five items while supporting a two-dimensional structure comprising psychological well-being and psychological distress.

The R-MHI-5 is particularly relevant because it provides an ultra-brief assessment of both positive and negative dimensions of mental health within the same instrument. This is consistent with contemporary approaches that conceptualize mental health as more than the absence of psychopathology and emphasize the joint assessment of well-being and distress. Its five-item format also reduces respondent burden, which is advantageous in adolescent, school, community, and large-scale assessment settings. This is especially relevant because instruments used to assess adolescent mental health frequently focus on specific aspects of well-being or psychological symptoms, whereas the R-MHI-5 allows both dimensions to be examined simultaneously (Orth et al., 2022; Rivera-Riquelme et al., 2019).

The R-MHI-5 has demonstrated adequate psychometric properties in adolescent populations, although the available evidence remains limited compared with that in adults. Rivera-Riquelme et al. (2019), in a sample of 595 Spanish children and adolescents aged 10 to 15 years, reported satisfactory psychometric properties for the R-MHI-5 and provided evidence supporting its use for the bidimensional assessment of psychological well-being and distress, as well as for screening symptoms of anxiety and depression. More recently, Rojas-Mendoza et al. (2024) examined the R-MHI-5 in 662 Peruvian adolescents aged 12 to 17 years. Their findings supported a two-dimensional structure comprising psychological well-being and psychological distress and provided adequate evidence of reliability. Despite these findings, psychometric studies of the R-MHI-5 in adolescent populations remain scarce, particularly in culturally and linguistically diverse populations.

The Spanish R-MHI-5 was selected as the source version for the present adaptation because it had already been linguistically adapted for Spanish-speaking children and adolescents and had subsequently shown psychometric evidence in the Peruvian context (Rivera-Riquelme et al., 2019; Vilca et al., 2022). Given that the aim of the present study was to adapt the R-MHI-5 for Southern Quechua-speaking adolescents in Peru, using the Spanish version provided continuity with the version already evaluated in the national context. Despite the available psychometric evidence, no culturally adapted version of the R-MHI-5 is currently available in Southern Quechua for adolescents from the Peruvian highlands. This gap may lead to inaccurate interpretations because, when the linguistic and cultural characteristics of Quechua-speaking adolescents are not adequately considered, their individual experiences of mental health may not be fully captured, potentially compromising appropriate assessment and intervention (Muñiz et al., 2013). Therefore, the aim of this study was to translate and culturally adapt the R-MHI-5 from Spanish into Southern Quechua and to examine its psychometric properties in adolescents residing in Angaraes, Huancavelica.

## Materials and methods design

This study aimed to translate and culturally adapt the R-MHI-5 into Southern Quechua and examine its psychometric properties. Accordingly, an instrumental, cross-sectional design was used (Ato et al., 2013).

### Participants

A total of 2,978 adolescents from the province of Angaraes, in the department of Huancavelica, Peru, participated in the study. The sample was selected through nonprobability convenience sampling across 14 educational institutions. The inclusion criteria were: (a) being between 10 and 19 years of age, (b) belonging to one of the selected educational institutions in the province of Angaraes, (c) being enrolled in secondary education, (d) being able to read and write in Southern Quechua, and (e) providing assent and parental informed consent. The age range corresponding to adolescence was established according to the classification of the World Health Organization (2020). The exclusion criteria included students with cognitive functional diversity, incomplete questionnaires, and those who were absent during the administration phase.

Participation was relatively balanced between girls (51.7%) and boys (48.3%). The mean age of the participants was 15 years, with an age range from 12 to 18 years. Regarding grade level, the distribution was similar across grades: first year (21.5%), second year (20.8%), third year (20.5%), fourth year (18.8%), and fifth year (18.4%). A total of 81.3% of the adolescents reported that they were not employed at the time of the study. Regarding parental status, 73.8% reported that their parents lived together, 18.8% that their parents were separated, 3.2% that their mother was widowed, 1.8% that they had never known their father, 1.6% that their father was widowed, 0.6% that they had never known either parent, 0.1% that they had never known their mother, and another 0.1% reported that both parents had died. Finally, 843 adolescents (28%) reported having experienced suicidal ideation at some point in their lives. In addition, 21.5%, 4.6%, 2.4%, and 3.3% reported having attempted suicide once, twice, three or four times, and five or more times, respectively.

### Instruments

#### Revised Mental Health Inventory-5 (R-MHI-5)

This is a brief instrument designed to assess mental health among adolescents and adults in the general population. It comprises two dimensions: psychological well-being (items 2 and 4) and psychological distress (items 1, 3, and 5), with the latter items reverse-worded. For both dimensions, the items refer to participants’ emotional states during the previous month and use Likert-type response options: never = 0, sometimes = 1, often = 2, and always = 3. In the present study, the version adapted to the Peruvian context was used, which had shown adequate psychometric properties for a two-dimensional factorial structure (CFI = 0.99, TLI = 0.99, RMSEA = 0.071) and adequate reliability for both psychological well-being (*ω* = 0.75) and psychological distress (*ω* = 0.79) (Vilca et al., 2022).

To translate the instrument into Southern Quechua, the back-translation method was used. A professional bilingual translator independently completed the initial translation from Spanish into Southern Quechua, while a second translator produced the back-translation from Southern Quechua into Spanish. Subsequently, both translators met with the study researchers to review and approve the final version. Five expert judges in clinical and educational psychology, with professional experience ranging from 6 to 35 years and all proficient in reading and writing Southern Quechua, were then invited to evaluate the items according to the criteria of relevance, representativeness, and clarity. Finally, before the formal administration of the instrument, a pilot test was conducted with seven Southern Quechua-speaking secondary school students from the selected educational institutions in Angaraes (four girls and three boys), aged 13 to 15 years. Participants completed the translated questionnaire and were subsequently asked whether they understood the wording of the items and whether they experienced any difficulties in understanding them. No comprehension difficulties or suggestions for modification were reported at this stage. The original Spanish version and its corresponding Southern Quechua translation are presented in Table 1.

**Table 1:** R-MHI-5 version translated from Spanish into Southern Quechua.

Items	Original Spanish version	Southern Quechua version
1	During the past month, how often have you felt very nervous?	Kay qipa killapi, ¿Hayka kutitataq sinchita mancharisqa sunquchakurqanki?
2	During the past month, how often have you felt calm and peaceful?	Kay ñawpaq killapi, ¿Hayka kutitan hawkalla qasilla sunquchakurqanki?
3	During the past month, how often have you felt discouraged or sad?	Qipa killapi, ¿Hayka kutitan hukmanyasqa utaq llakisqa sunquchakurqanki?
4	During the past month, how often have you felt happy?	Qipa killapi, ¿Hayka kutitan kusisqa sunquchakurqanki?
5	During the past month, how often have you felt so sad that nothing could cheer you up?	Qipa killapi, ¿Hayka kutitan sinchi llakisqa sunquchakurqanki, manataqmi imapas kusichisunkimanchu?

### Procedure

Data were collected in person at 14 full-school-day educational institutions (JEC) under the Local Educational Management Unit (UGEL) of Angaraes Province, Huancavelica. These institutions provided secondary education only, and a formal request for authorization was submitted to each school principal. Subsequently, the research team coordinated with tutoring coordinators and teaching staff to establish schedules and classroom access. Data collection took place between March and April 2024.

All participants were given a brief explanation of the purpose of the study and the ethical principles governing the research process, including anonymity of responses, confidentiality of information, and voluntary participation. Before the questionnaires were distributed, students submitted informed consent forms signed by their parents or legal guardians, as well as their own assent. During questionnaire administration, the research team answered participants’ questions, and they were reminded that they could withdraw from the study at any time if they wished.

### Statistical analysis

Statistical analyses were conducted in several phases using RStudio version 4.3.2. In the first phase, descriptive analyses of the study population were performed using absolute and relative frequencies. For the items, the mean, standard deviation, skewness, kurtosis, corrected item–test correlation, and polychoric correlation matrix were calculated using the psych package (version 2.6.5). Corrected item–test correlations greater than 0.20 were considered acceptable (Streiner et al., 2024). Polychoric correlations were used because the items were measured using ordered categorical response options (Marôco, 2024). Content validity was assessed using Aiken’s *V* coefficient, with values greater than 0.70 considered acceptable (Roebianto et al., 2023).

In the second phase, confirmatory factor analysis (CFA) was performed using the lavaan package (version 0.7-2) with the robust weighted least squares mean- and varianceadjusted estimator (WLSMV). Model adequacy was evaluated using fit indices such as the Comparative Fit Index (CFI > 0.95), Root Mean Square Error of Approximation (RMSEA < 0.08), and Standardized Root Mean Square Residual (SRMR < 0.08) (Jordan Muiños, 2021). In the third phase, reliability was estimated using the Bayesian omega coefficient (*ω*), H coefficient, and empirical reliability (rxx). Bayesian omega was estimated using the Bayesrel package (version 0.7.9) with the default prior specifications for the single-factor model: normal priors centered at zero for the factor loadings and factor scores, an inverse-gamma prior with shape = 2 and scale = 1 for the residual variances, and an inverse-Wishart prior for the latent factor variance. The posterior mean and the 95% highest posterior density credible interval were reported. The H coefficient was calculated using a custom R function based on the standardized factor loadings obtained from the CFA model, following $H={\left[1+1/\sum (\lambda_{i}^{2}/(1-\lambda_{i}^{2}))\right]}^{-1}$ (Hancock and Mueller, 2001). Empirical reliability was estimated using the mirt package (version 1.47) (Baños-Chaparro and Caycho-Rodríguez, 2024; Du Toit, 2003; Gunnell, 2020). Values ≥ 0.70 were considered indicative of acceptable reliability. For Bayesian omega, this criterion was applied to the posterior estimate, while the 95% highest posterior density credible interval was also considered to reflect the uncertainty of the estimate (Baños-Chaparro and Caycho-Rodríguez, 2024; Pfadt et al., 2022). For coefficient H, this threshold was based on the criterion proposed by Hancock and Mueller (2001) for adequate construct replicability. Empirical reliability (rxx) was interpreted using the same conventional lower bound of 0.70 for reliability coefficients within the IRT framework (Sung and Liu, 2026).

Subsequently, in the fourth phase, measurement invariance was examined by sex because sex-related differences in adolescent mental health are well documented and girls and boys were approximately equally represented in the sample. Factorial invariance by sex was evaluated using the semTools package (version 0.5-9) by hierarchically constraining the model across configural, threshold, metric, scalar, and strict levels. Changes in fit indices were considered acceptable when ΔCFI < 0.010, ΔRMSEA < 0.015, and ΔSRMR < 0.030 (Chen, 2007).

In the fifth phase, a two-parameter item response theory (IRT) model was estimated using the mirt package (version 1.47): the discrimination parameter (a), which distinguishes between individuals with different levels of the latent trait (*θ*), with values greater than 1 considered acceptable (Baker and Kim, 2017); and the difficulty parameter (β), which indicates the probability of selecting among different response options according to the level of the trait. The graded response model (GRM) was estimated. The latent trait (*θ*) was expressed on a standardized metric with a mean of 0 and a standard deviation of 1, where negative values indicate lower levels of the latent trait, values around 0 represent average levels, and positive values indicate higher levels. The assumptions of unidimensionality, local independence using the standardized LD-*χ*^2^ statistic (< 10), with the mirt package (version 1.47), and monotonicity using the non-parametric Mokken model with the mokken package (version 3.1.2) (Sijtsma and van der Ark, 2017; Stover et al., 2019). Item information curves (IICs) and the test information curve (TIC) were also examined to estimate measurement precision across levels of *θ*.

### Ethical considerations

The study was approved by the Ethics Committee of Universidad para el Desarrollo Andino under registration No. II-UDEA-2024-001. All participants provided their assent, together with informed consent signed by their parents or legal guardians. National and international ethical guidelines in the field of psychology were also followed, ensuring participants’ anonymity, voluntary participation, protection of the information collected, and the right to withdraw from the study at any time without any consequences (Knapp and VandeCreek, 2003).

## Results content-based evidence

The expert judges evaluated the content of the items according to the criteria of relevance, representativeness, and clarity, yielding Aiken’s *V* values above 0.70 (Table 2). The final version of the R-MHI-5 translated into Southern Quechua was approved by all judges.

**Table 2:** Content validity evidence for the R-MHI-5 items.

Items	Relevance (*n* = 5)		Representativeness (*n* = 5)		Clarity (*n* = 5)	
	*V*	CI 95%	*V*	CI 95%	*V*	CI 95%
1	0.80	0.59, 0.92	0.87	0.67, 0.95	0.93	0.75, 0.99
2	0.87	0.67, 0.95	0.73	0.52, 0.87	0.80	0.59, 0.92
3	0.87	0.67, 0.95	0.93	0.75, 0.99	0.73	0.52, 0.87
4	0.73	0.52, 0.87	0.80	0.59, 0.92	0.93	0.75, 0.99
5	0.93	0.75, 0.99	0.73	0.52, 0.87	0.80	0.59, 0.92

*V*, Aiken’s *V*, CI, Confidence intervals.

### Descriptive analysis

Item 4 showed the highest mean and standard deviation, whereas item 1 presented the lowest values (Table 3). Skewness values ranged from −0.05 to 1.20, while kurtosis values ranged from −1.29 to 0.84. Corrected item–test correlations ranged from 0.29 to 0.37, with all values exceeding the established criterion of 0.20. Polychoric correlations were positive and ranged from 0.09 to 0.63 (Table 3).

**Table 3:** Descriptive measures and correlation matrix.

Items	*M*	SD	*g*_1_	*g*_2_	*r*_it_	Correlation matrix				
1	1.49	0.65	1.13	0.77	0.37	–				
2	2.30	1.06	0.32	−1.11	0.36	0.16	–			
3	1.73	0.81	0.90	0.14	0.36	0.58	0.12	–		
4	2.56	1.09	−0.05	−1.29	0.37	0.16	0.63	0.15	–	
5	1.60	0.78	1.20	0.84	0.29	0.47	0.10	0.52	0.09	–

*M*, mean; SD, standard deviation; *g*_1_, skewness; *g*_2_, kurtosis; *r*_it_, corrected item– test correlation.

#### Evidence based on internal structure

The two-dimensional factorial structure, comprising psychological distress and psychological well-being, showed adequate fit, with favorable indices (CFI = 0.99, RMSEA = 0.034 [90% CI: 0.031, 0.047], and SRMR = 0.01) and factor loadings above 0.60 (Figure 1).

**Figure 1:**
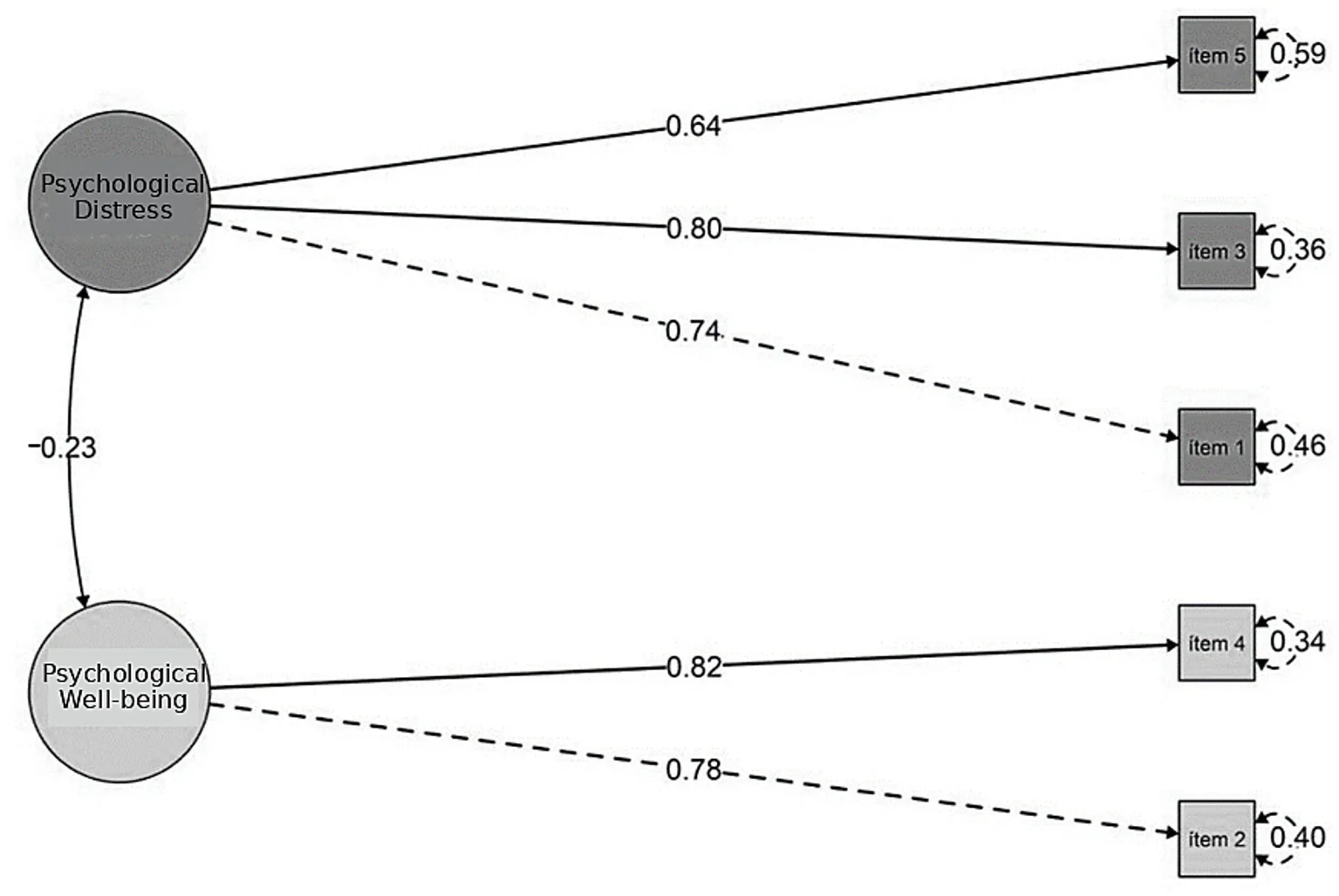
Factor structure of the R-MHI-5. Values represent standardized factor loadings and the standardized correlation between the latent factors. Solid lines represent freely estimated parameters, whereas dashed lines represent factor loadings fixed for model identification.

#### Internal consistency

The posterior mean of the *ω* coefficient was 0.72 for both psychological distress and psychological well-being, with a 95% highest posterior density credible interval of 0.69 to 0.74. In addition, the H coefficient was acceptable for both psychological distress (*H* = 0.78) and psychological well-being (*H* = 0.78). Empirical reliability was acceptable for psychological well-being (rxx = 0.71), whereas the estimate for psychological distress (rxx = 0.66) was slightly below the conventional 0.70 criterion.

#### Measurement invariance

Table 4 presents the results of the factorial invariance analysis. The configural model did not differ substantially from the more restrictive models, as changes in fit indices remained within acceptable limits (ΔCFI < 0.010; ΔRMSEA < 0.015; ΔSRMR < 0.030). These findings support measurement invariance of the R-MHI-5 across girls and boys.

**Table 4:** Measurement invariance of R-MHI-5 by sex.

Models	*χ*^2^ (df)	*p*	CFI	RMSEA	SRMR	ΔCFI	ΔRMSEA	ΔSRMR
M1	19.64 (8)	0.012	0.997	0.031	0.016	–	–	–
M2	25.33 (13)	0.021	0.997	0.025	0.016	0.000	0.006	0.000
M3	25.25 (16)	0.065	0.998	0.020	0.018	0.001	0.006	0.001
M4	39.11 (19)	0.004	0.995	0.027	0.022	0.003	0.007	0.004
M5	68.87 (24)	0.000	0.990	0.035	0.025	0.006	0.009	0.003

*χ*^2^, chi-square; df, degrees of freedom; M1, configural invariance; M2, Threshold invariance; M3, Metric invariance; M4, Scalar invariance; M5, Strict invariance.

#### Item response theory

The assumptions were supported by the unidimensional CFA results for both psychological well-being and psychological distress. Local independence assessed through LD-*χ*^2^ showed standardized relationships below 10 (range = −0.058 to 0.085), and no significant violations of monotonicity were identified (crit < 40). Regarding the a parameter, items 4 and 3 showed the highest discrimination values for the psychological well-being and psychological distress dimensions, respectively. With respect to the β parameters, the probability of endorsing successive response categories increased consistently across levels of *θ* (Table 5). As shown in Figure 2, item 4 for psychological well-being and item 3 for psychological distress provided the greatest amount of item-level information. The test information curves indicated greater measurement precision around the center of the *θ* distribution for psychological well-being and at positive *θ* values for psychological distress.

**Table 5:** Discrimination, difficulty, and item-fit parameters.

Dimensions	Item parameters				
	Items	*a*	*β*1	*β*2	*β*3
Psychological wellbeing	2	2.17	−0.804	0.345	1.142
	4	2.53	−0.959	−0.038	0.794
Psychological distress	1	1.96	0.307	1.964	3.452
	3	2.27	−0.092	1.256	2.361
	5	1.52	0.213	1.738	2.965

*a*, discrimination parameter; *β*, difficulty parameter.

**Figure 2:**
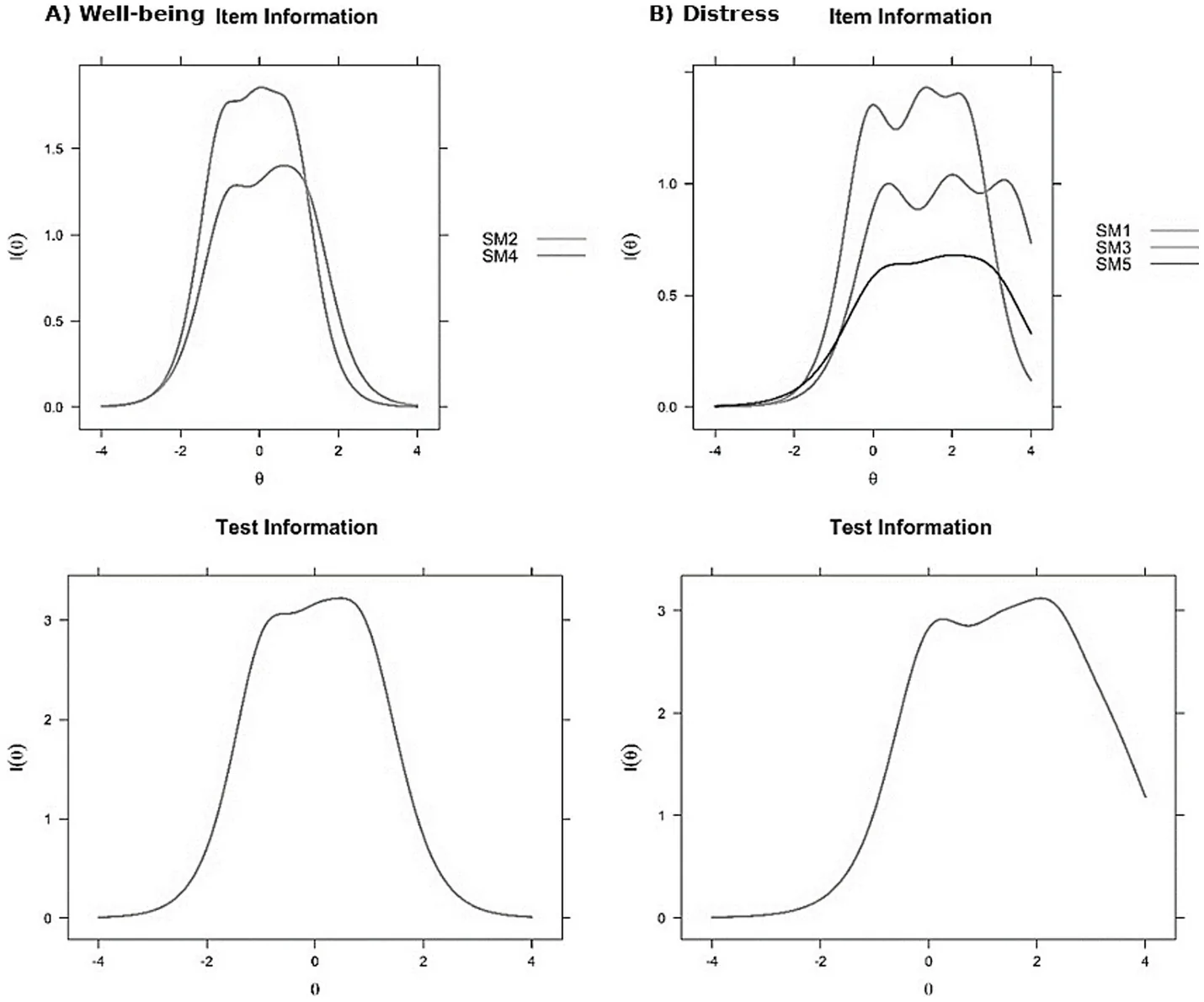
Item **(A)** and test **(B)** information functions of the R-MHI-5. SM1–SM5 correspond to items 1–5 of the R-MHI-5. SM2 and SM4 assess psychological wellbeing, whereas SM1, SM3, and SM5 assess psychological distress. *I*(*θ*) represents information across levels of the standardized latent trait (*θ*), where *θ* = 0 represents the center of the latent trait distribution.

## Discussion

Assessing mental health in adolescents is essential for the design of prevention and early intervention programs in healthcare and community settings (WHO, 2024). The availability of brief, reliable, and culturally appropriate instruments can complement psychological assessment and facilitate research in this population. The findings of this study indicate that the R-MHI-5 translated into Quechua has adequate psychometric properties for assessing mental health in adolescents. Factor analysis confirmed a two-dimensional structure comprising psychological well-being and psychological distress, with satisfactory reliability and measurement invariance by sex, supporting its usefulness as a brief mental health assessment instrument.

The factorial structure identified is consistent with findings reported in Peruvian adolescents from urban areas (Rojas-Mendoza et al., 2024) and Spanish adolescents (Rivera-Riquelme et al., 2019), suggesting that the two-dimensional model of well-being and distress is maintained across different languages and sociocultural contexts. Although it has been argued that instruments developed in Western countries may not fully capture the experiences of young people from other cultures (Orth et al., 2022), evidence from Quechua-speaking adolescents showed that they also consistently recognize both dimensions. This supports the dual model of mental health (Greenspoon and Saklofske, 2001; Magalhães, 2024), which proposes that simultaneously considering well-being and distress makes it possible to identify profiles such as complete mental health (high well-being and low distress), vulnerable (low well-being and low distress), symptomatic but content (high well-being and high distress), and troubled (low well-being and high distress) (Suldo and Shaffer, 2008; Antaramian et al., 2010; Magalhães and Calheiros, 2017). Notably, the vulnerable and symptomatic-but-content profiles are often overlooked in research and clinical practice despite their relevance (Magalhães, 2024). Therefore, having the R-MHI-5 available in Quechua may facilitate the identification of these profiles, reduce the risk of underdetection, and guide differentiated interventions among adolescents.

In addition, the Quechua version of the R-MHI-5 showed generally satisfactory reliability evidence. Bayesian omega and H coefficients met the conventional criterion for both dimensions, whereas empirical reliability was acceptable for psychological well-being and slightly below the conventional threshold for psychological distress. From an IRT perspective, all items showed satisfactory psychometric performance. Item 4 showed the highest discrimination for psychological well-being, whereas item 3 showed the highest discrimination for psychological distress. These findings are partly consistent with those reported by Vilca et al. (2022) in a Peruvian Spanish-speaking sample, where item 4 was the most accurate item for assessing psychological well-being, while items 3 and 5 showed the greatest accuracy for psychological distress. Thus, the strong performance of items 4 and 3 appears to be relatively consistent across the Spanish and Southern Quechua versions of the R-MHI-5. In Peruvian adolescents, Rojas-Mendoza et al. (2024) also found that item 3.

had the highest network loading within the psychological distress dimension, although these results are not directly comparable because that study used network psychometrics rather than IRT. The difficulty parameters followed the expected ordering across response categories. The test information curves indicated greater measurement precision around the center of the *θ* distribution for psychological well-being and at positive *θ* values for psychological distress. Taken together, these findings support the brevity, efficiency, and usefulness of the instrument as a brief measure of psychological well-being and psychological distress.

During adolescence, mental health differences by sex have been observed, with adolescent girls tending to report more symptoms of depression and anxiety than boys (Yoon et al., 2023). In this study, the measurement invariance results indicated that the scale functions equivalently across boys and girls, supporting meaningful comparisons between boys and girls and indicating that observed group differences are less likely to reflect measurement bias. This finding is particularly relevant in rural Andean communities, where gender roles are often shaped by cultural norms that establish different expectations regarding behavior and emotional expression for boys and girls (Kang, 2010), and where Quechua-speaking women have historically faced inequalities (Huamán, 2019). Thus, the evidence of invariance supports the usefulness of the instrument for comparisons by sex among Quechua-speaking adolescents.

Beyond sex-related differences, rural Andean communities also face disadvantages such as limited access to healthcare, economic constraints, and social and cultural factors that widen existing inequalities (Pan American Health Organization [PAHO], 2021). In addition, stigma surrounding mental health services and limited community support may negatively affect well-being (Moya-Salazar et al., 2023). These challenges are compounded by a history of conflict and violence with long-lasting consequences (Román et al., 2019). In this context, having psychometrically sound and culturally appropriate instruments, such as the Quechua version of the R-MHI-5, is important for assessing and monitoring mental health in this historically underserved population.

These challenges are further compounded by linguistic barriers: many healthcare professionals do not speak Quechua (Montesinos-Segura et al., 2018), and bilingual family members sometimes serve as interpreters during assessments, a practice that may introduce bias (Cjuno et al., 2023). Therefore, having instruments validated in the native language addresses an important gap in psychological assessment and is essential for providing mental health care that is understandable and accessible (Vecchio et al., 2022; Carranza Esteban et al., 2024).

Despite these contributions, several limitations should be acknowledged. The sample consisted of school-attending adolescents from a single province located within the Chanka-speaking area of Southern Quechua. Therefore, the findings should not be assumed to generalize to speakers of other Southern Quechua varieties, such as Collao Quechua, or to Northern, Central, or Amazonian Quechua. Although some Quechua varieties share linguistic features, further cultural adaptation and psychometric evaluation are needed before the R-MHI-5 can be used with speakers of these varieties. The crosssectional design prevented the assessment of temporal stability, and the lack of external measures precluded the assessment of convergent and criterion validity, sensitivity and specificity, and the establishment of clinical cutoff scores. Therefore, the present findings should not be interpreted as evidence supporting the diagnostic or clinical screening accuracy of the Southern Quechua R-MHI-5. Future studies should incorporate clinical interviews and external criterion measures to examine these properties.

Overall, the validation of the R-MHI-5 in Southern Quechua provides a brief, culturally appropriate, and easy-to-administer tool for community, healthcare, and research settings in the Andean region. Its five-item format facilitates rapid assessment in resource-limited settings and, by being available in the native language, may help reduce longstanding linguistic barriers to valid assessment among Quechua-speaking adolescents. At the same time, the findings consistently support the dual model of well-being and distress, reinforcing the complete mental health model and indicating that this two-dimensional structure is also supported in cultural and linguistic contexts different from those in which the instrument was originally developed. Thus, the Quechua version of the R-MHI-5 represents progress toward more inclusive assessment practices that are better adapted to the realities of Quechua-speaking communities. It also helps identify profiles of psychological well-being and distress that might otherwise remain undetected in traditional assessment systems.

In conclusion, the Southern Quechua version of the R-MHI-5 showed satisfactory psychometric properties, including adequate internal consistency, measurement invariance by sex, and satisfactory item functioning. Its validation represents an important contribution to brief and culturally appropriate mental health assessment among Quechua speaking adolescents, a historically underserved population. The availability of this instrument facilitates brief mental health assessment in community and healthcare settings with limited resources, may help reduce linguistic barriers, and helps identify wellbeing and distress profiles that are often overlooked. These findings support the usefulness of the R-MHI-5 as a brief instrument for research and mental health monitoring among Quechua-speaking adolescents.

## Data Availability

The raw data supporting the conclusions of this article will be made available by the authors, without undue reservation.
